# Seizure Following Zolpidem Withdrawal in a Patient Presenting as a Stroke Lysis Call: A Case Report

**DOI:** 10.7759/cureus.94684

**Published:** 2025-10-15

**Authors:** Hadi Chishti, Eoin Melby, Zainab Mansoor

**Affiliations:** 1 Internal Medicine, Ulster Hospital, Belfast, GBR; 2 Medicine, Ulster Hospital, Belfast, GBR

**Keywords:** acute stroke mimic, gamma-aminobutyric acid, seizures, stroke, substance-related disorders, zolpidem

## Abstract

Stroke lysis, or thrombolysis, is a time-sensitive intervention for acute ischemic stroke, but careful distinction from stroke mimics is essential to avoid inappropriate treatment. Zolpidem, a non-benzodiazepine hypnotic, is generally regarded as having low dependence potential; however, prolonged or high-dose use can result in withdrawal syndromes, including seizures. We report the case of a 46-year-old woman presenting with acute left upper limb weakness and facial sensory changes, initially managed as a stroke lysis call. She was outside the lysis window and had initial negative neuroimaging. Within 24 hours, she developed recurrent generalized seizures requiring intravenous lorazepam. Subsequent collateral history revealed chronic high-dose zolpidem use (50-200 mg daily), discontinued abruptly, confirming a diagnosis of zolpidem withdrawal seizure. The patient was successfully treated with a benzodiazepine taper and discharged with community mental health and substance misuse follow-up. This case emphasizes the importance of thorough medication history-taking in stroke evaluations, as withdrawal syndromes can closely mimic acute cerebrovascular events. It is also interesting to note that zolpidem can increase the risk of strokes.

## Introduction

Zolpidem is a short-acting hypnotic of the imidazopyridine class that selectively binds to the GABA-A receptor α1 subunit, producing sedative effects. It is prescribed for short-term management of insomnia, with guideline recommendations limiting its use to four weeks at a nightly dose of 5-10 mg ​[1].​

Although initially considered to have lower abuse potential than benzodiazepines, increasing evidence indicates that chronic or high-dose zolpidem use can lead to dependence and withdrawal syndromes. Reported withdrawal symptoms range from anxiety, palpitations, and tremors to delirium and generalized seizures ​[2,3]. ​ 

We describe a patient who developed recurrent generalized seizures following abrupt discontinuation of high-dose zolpidem. Her initial presentation as a suspected acute ischemic stroke highlights the diagnostic challenge posed by withdrawal syndromes in acute stroke pathways. 

## Case presentation

A 46-year-old female, with no significant co-morbidities, presented to her local Emergency Department with a four-hour history of left upper limb weakness, reduced grip strength, and reduced sensation on the left side of her face. When clarifying her history, she stated having felt generally unwell for several days, but no infectious symptoms. Her past medical history included anxiety and hypertension. She was on once nightly zopiclone and atorvastatin/irbesartan for primary prevention. On examination, she did indeed have left upper limb weakness and reduced grip, but there were no eye symptoms, gait abnormalities, cranial nerve defects, or sensory loss. There was nothing of note on examination of reflexes or coordination. She was initially treated as a Stroke Lysis Call, but she was found to be outside the lysis window and was not lysed. Her CT Brain/Angiogram did not show any acute findings. She was loaded with Aspirin as per stroke protocol and planned for an inpatient MRI. There was nothing outstanding regarding her blood results. Within the next 24 hours, the patient proceeded to have two “seizure” type episodes, which resolved with IV lorazepam. Both times, the patient slid off the bed and had multiple limb jerking actions. She had one episode where she bit her tongue, but did not have any incontinence. Lactate levels were raised to 11 mmol/L at the time (normal range 0.5-2.2 mmol/L). The neurology team advised videotaping any further seizure activity. 

Finally, when talking to her family members, it was revealed that she had tried to access >600mg of Zolpidem, purchased privately. This had not been established from initial history taking and successive daily reviews. Later on, the patient told medical staff that she had been taking 50-200mg of zolpidem daily, which she self-titrated up. She also informed medical staff that she was taking varying amounts of these tablets at different times of day when she felt unwell. When liaising with substance misuse and mental health teams, it became apparent that the patient had been taking excessive amounts of zolpidem for sleep relief, and she had stopped this practice abruptly. Further, there were no acute findings on her MRI scan, which confirmed a diagnosis of Z-drug withdrawal. The patient was managed well on weaning dose chlordiazepoxide, and having been linked in to community mental health and substance misuse teams, was suitable for discharge. This patient had insight into her experiences and is now keen for others to be aware of similar symptoms. This case reinforces the importance of taking a good medical history and highlights the importance of stroke mimics.

## Discussion

Although zolpidem is marketed as safer than benzodiazepines, dependence and withdrawal complications are increasingly recognized. Reports describe withdrawal syndromes, including tremors, delirium, and seizures, even with moderate doses ​[2,3]. Our patient’s case highlights how abrupt zolpidem withdrawal can present as a stroke mimic, delaying diagnosis and management.

Stroke mimics represent nearly one-third of suspected acute stroke cases ​[4]. In this case, focal neurological deficits and acute onset symptoms initially prompted a lysis call, but subsequent seizures and detailed collateral history revealed the true diagnosis. This underscores the importance of careful history-taking and collateral information in stroke evaluations. Recent literature reviews estimate an increasing prevalence of zolpidem misuse, dependence, and abuse ​[5]. In one of the studies the most common causes of stroke mimics were peripheral vestibular disorder (27.4%), psychogenic (24.4%), seizure (9.2%), migraine (9.2%), and metabolic and drugs (9.2%) [6].

The pathophysiology involves adaptive changes in GABA-A receptor function after chronic exposure, resulting in hyperexcitability upon abrupt cessation ​[7]. Reported management strategies include acute seizure control with intravenous benzodiazepines and gradual tapering with long-acting benzodiazepines such as diazepam or chlordiazepoxide ​[8,9]. Our patient’s successful treatment followed this approach. 

Recent literature further supports the severity of zolpidem dependence. Moshfeghinia et al. (2023) described a patient with over 10 years of chronic high-dose zolpidem use, presenting with severe withdrawal manifestations, reinforcing the drug’s potential for long-term dependence and highlighting the need for careful prescribing and monitoring ​[10]. Together with our case, these reports stress that zolpidem withdrawal can be considered in patients with unexplained seizures or atypical neurological presentations if there is a history of zolpidem use or abuse. 

This case reinforces three clinical lessons, which include that zolpidem withdrawal should be considered in patients presenting with acute neurological deficits or seizures, a detailed medication history is vital in differentiating stroke from mimics, and multidisciplinary involvement, including neurology and psychiatry, ensures safe withdrawal management and relapse prevention. What makes it a bit complex is that zopidem actually increases the risk of strokes as per initial research, but more research is needed in this area [11].

## Conclusions

Zolpidem withdrawal can precipitate seizures that mimic acute stroke, leading to potential misdiagnosis and inappropriate management. A thorough drug history, particularly regarding hypnotics and sedatives, is essential in acute stroke pathways. Early recognition of withdrawal syndromes, prompt seizure control with benzodiazepines, and structured follow-up with mental health and addiction services are key to improving patient outcomes. 
